# Does longer duration of corticosteroid treatment improve clearance in vulvar lichen sclerosus? Results from a single centre, comparative, open label study

**DOI:** 10.1111/dth.14955

**Published:** 2021-05-04

**Authors:** Monica Corazza, Giulia Toni, Giorgia Valpiani, Chiara Morotti, Alessandro Borghi

**Affiliations:** ^1^ Department of Medical Sciences, Section of Dermatology and Infectious Diseases University of Ferrara Ferrara Italy; ^2^ Research Innovation Office S. Anna University Hospital of Ferrara Ferrara Italy

**Keywords:** active treatment phase, effectiveness, mometasone furoate, tolerability, treatment duration, vulvar lichen sclerosus

## Abstract

A complete clearance of vulvar lichen sclerosus (VLS) is achieved in a minority of patients treated with a standard 12‐week duration corticosteroid treatment. The aim of this pragmatic, retrospective, open label, comparative trial was to assess the effectiveness, in terms of complete clearance, of a 24‐week treatment with mometasone furoate 0.1% ointment (MMF) and to compare it with a 12‐week therapy. We included VLS patients treated with MMF administered for five consecutive days/week for 24 weeks (group A). The following were assessed: (a) clearance in Global Subjective Score (GSS), Global Objective Score (GOS) or both, (b) changes of these parameters and dyspareunia at treatment completion compared to baseline, (c) safety profile. All these assessments were compared with the same outcomes recorded among VLS patients who had previously undergone a 12‐week MMF treatment (group B). Twenty‐nine patients were included in group A and 32 in group B. The rates of patients who achieved the clearance of GSS, GOS or both parameters did not significantly differ between groups A and B. The groups did not differ in any of the effectiveness outcomes assessed. A 24‐week duration corticosteroid treatment does not seem to provide significant therapeutic benefits in comparison with standard 12‐week courses, especially considering the occurrence of complete clearance.

## 1 INTRODUCTION

The ideal treatment for vulvar lichen sclerosus (VLS) should aim at inducing relief of symptoms, reversing signs and preventing further anatomical changes.^1^ Adherence to long‐term treatment was also shown to prevent progression towards cancer.^2^, ^3^ On the basis of the immunological pathophysiology of VLS, high potency topical corticosteroids represent the first‐line treatment in the active phase of the disease.^1^, ^4^, ^5^ Consistent with this, there is strong evidence that potent and ultra‐potent corticosteroids have an excellent effect on both symptom control and, although to a lesser extent, on reversal of clinical signs.^6^, ^7^, ^8^, ^9^ This evidence is largely based on trials of 12 week‐duration,^6^, ^7^, ^8^, ^9^, ^10^, ^11^, ^12^, ^13^, ^14^ which is the conventional duration of trials assessing the effectiveness of topical treatments in the active phase of the disease.

Despite significant improvement in symptoms and signs, a recent retrospective study highlighted the fact that complete cure of VLS occurs in a minority of patients treated with 12‐week therapies.^15^ This means that, at treatment completion, most patients may still have substantial residual disease, with possible negative effects on their well‐being and quality of life. In this regard, one might wonder if treatment courses that are longer than the conventional duration may provide therapeutic advantages and increase the chance of complete clearance.

The present study purposely assessed effectiveness and tolerability of a 24‐week treatment with a potent corticosteroid. A comparison was also made with the treatment outcomes achieved with a standard 12‐week duration therapy with the same molecule.

## 2 MATERIALS AND METHODS

### 2.1 Study design and objectives

The present study was conducted between March 2019 and March 2020 (enrollment period) at the Vulva Unit of the University Hospital of Ferrara, Italy. The study was set up as a single‐centre, pragmatic, retrospective, open label, comparative trial. The aims of the study were: (a) to assess the effectiveness and tolerability of mometasone furoate (MMF) 0.1% ointment in a 24‐week treatment of active VLS (group A); (b) to assess the rate of patients who either discontinued the treatment or did not attend the control visit; (c) to retrospectively compare the results of the study therapy with those achieved with the same glucocorticoid molecule, administered with the same treatment regimen, but over a 12‐week duration (group B).

This was a spontaneous trial with no funding from external sources. The principles outlined in the Helsinki Declaration of 1975, as revised in 1983, were followed for all the patients treated at our outpatient vulvar Clinic. The study was approved by the University‐Hospital of Ferrara institutional review board (CE: 827/2020/Oss/AOUFe). Patients provided their written informed consent.

### 2.2 Study patients

During a 6 month‐duration inclusion phase, we treated all the adult (≥18 years) female patients affected with VLS with MMF 0.1% ointment, administered for five consecutive days a week, for 24 weeks. Among these patients, those with clinical and histological diagnosis of VLS were eligible (group A). Patients were excluded from the study in the presence of the following: lack of histological confirmation; clinical or histological features showing possible resemblance with other diseases, such as lichen planus or plasma cell vulvitis; lack of agreement between clinical and histological features; topical and/or systemic VLS treatments during the 8 weeks before enrollment; MMF administered with treatment regimens or at formulations different from those considered in the study (see below); active vulvar infectious diseases, confirmed by cultural specimens, or vulvar carcinoma, histologically proven; pregnancy or breastfeeding.

### 2.3 Study procedures and assessments

All the study subjects were instructed to apply a pea‐sized quantity of MMF 0.1% ointment on the affected vulvar surfaces once daily for five consecutive days a week for the entire treatment duration. Throughout the treatment duration no additional local or systemic treatments, nor cosmetics expected to relieve VLS, were administered.

Subjective evaluation of the following two symptoms, itching and burning, was obtained by interview using a visual analogue scale (VAS, which includes a numeric rating scale 0‐10). A global subjective score (GSS) was obtained by summing the scores of each symptom parameter (highest GSS = 20). Dyspareunia (VAS 0‐10) was considered separately from the other symptoms, as it was not evaluated in a considerable part of study patients who avoided sexual activity for reasons other than disease‐related pain.

The following four objective parameters were considered in order to evaluate the clinical features of the disease at baseline and to monitor VLS response to treatment: (a) leukoderma (pallor), (b) erythema, (c) hyperkeratosis, (d) purpuric lesions and itching‐related excoriations. Objective assessment of each sign was performed by the investigators using the following 4‐point scale: 0 = absence, 1 = mild, 2 = moderate, 3 = severe. A global objective score (GOS) was obtained by summing the scores of each clinical parameter (highest GOS = 12). Sclerosis‐scarring was not included in the GOS as it is not reversible to therapy, or only scarcely so.

Objective and subjective patient assessment was performed in consensus by the same experienced investigators at baseline and at the 24‐week control visit.

The following data were recorded by interview through a verbally administered questionnaire: (a) age at the beginning of treatment; (b) age at the onset of VLS, taken as the age when the women first experienced VLS‐related symptoms; (c) age at diagnosis, defined as the age when clinical or histological diagnosis was established; (d) disease duration, recorded as the time between the patient‐reported onset of symptoms and beginning the treatment; (e) previous therapy with topical corticosteroids, defined as documented courses with corticosteroids of at least 12‐week duration prior to being included.

### 2.4 Main outcome measures

The primary study endpoint was to assess the rate of patients achieving complete clearance in symptoms, that is, GSS = 0, in objective features, GOS = 0, and in both scores, that is GSS and GOS = 0.

The secondary effectiveness outcomes were: (a) the variation in the median values of GSS, dyspareunia and GOS at treatment completion compared with baseline, (b) the rate of patients who achieved GSS75, D75, and GOS75, which corresponds to an improvement from baseline of ≥75% in the GSS, dyspareunia and GOS values, respectively.

The tolerability profile of the treatment was assessed by recording any side effects experienced by the study patients.

The rate of dropouts over the treatment period was also addressed. Dropouts included both patients who discontinued treatment for any reason other than the occurrence of side effects and those who did not attend the control visit. This can serve as a measure of feasibility of a prolonged treatment.

### 2.5 Comparison with a 12‐week duration treatment

All the study effectiveness, tolerability, and dropout assessments were compared with the same outcomes recorded among adult patients with a histologically proven VLS who had undergone a 12‐week treatment with MMF 0.1% ointment applied once daily for five consecutive days a week, during the 9 months before the study period (June 2018‐February 2019, group B). Patients treated with other corticosteroid molecules, or with MMF used with alternative regimens or formulations, were excluded. Patients were also excluded from this retrospective inclusion if any single baseline data necessary for our analysis was incomplete. No further selection criteria were adopted, in order to minimize possible comparative biases.

### 2.6 Statistical analysis

The Shapiro‐Wilk test was used to assess the normality of distribution of the continuous variables. In the presence of symmetry of the distributions, the variables were represented with mean and SD or, in the case of non‐normal distribution, with the median value and interquartile range [1Q 3Q]; categorical data were expressed as total numbers and percentages.

In order to assess whether the two groups were comparable at inclusion, the Student *t* test was used for analyzing the variables “age at the VLS onset” and “age at the beginning of treatment,” the Mann–Whitney test was used for the variables “GSS,” “GOS,” “dyspareunia,” and “disease duration” and the Chi square test for the following: “previous therapy with topical corticosteroids” and “patients evaluable for dyspareunia.” Chi‐square test or Fisher's exact test, depending on the minimal expected count in each crosstab, was used to identify if there were differences between groups in the rates of subjects: (a) who reached a score equal to 0 for GSS, GOS or GSS and GOS, (b) who dropped out and (c) who had a reduction ≥75% for GSS, GOS or dyspareunia. The Mann–Whitney test was used to analyze the differences in GSS, GOS and dyspareunia variations between group A and group B patients. All analyses were performed using Stata 15.1 SE (Stata Corporation, College Station, Texas, USA). *P* value <.05 was defined as statistically significant.

## 3 RESULTS

### 3.1 Patient characteristics

Twenty‐nine patients were included in group A and 32 in group B. Demographic and clinical data of the VLS patients included are reported in Table 1. All demographic and disease features resulted well balanced between the two groups, except for dyspareunia, which had a higher median value in group A patients than those in group B.

**TABLE 1:** The study patients' characteristics at baseline

Variables	Group A 24 week‐duration (total, n. 29)	Group B 12 week‐duration (total, n. 32)	*P*‐value
Age at inclusion, years, mean (SD) [range]	63.89 (9.69) [42‐86]	62.53 (11.68) [40‐86]	0.623
Age at VLS onset, years, mean (SD) [range]	59.31 (11.94) [28‐85]	58.75 (12.17) [30‐83]	0.857
Disease duration, years, median [1Q 3Q]	1 [1 5]	2 [1 5]	0.947
Previous therapy with topical corticosteroids^a^			0.913
Yes, n. (%)	15 (51.7)	17 (53.1)	
No, n. (%)	14 (48.3)	15 (46.9)	
Patients evaluable for dyspareunia			0.395
Evaluable, n. (%)	15 (51.7)	20 (62.5)	
Not evaluable, n. (%)	14 (48.3)	12 (37.5)	
Global Subjective Score (0‐20) median [1Q 3Q]	14 [7 20]	12 [5.5 16]	0.218
Itching (0–10), median [1Q 3Q]	6 [2 9]	8 [5 9.5]	0.478
Burning (0–10), median [1Q 3Q]	4 [0 10]	5.5 [0 8]	0.504
Dyspareunia^a^ (0‐10), median [1Q 3Q]	10 [0 10]	2.5 [0 7.5]	**0.039**
Global Objective Score (0‐12), median [1Q 3Q]	4 [3 5]	4 [3 5.5]	0.351
Erythema, median [1Q 3Q]	1 [0 1]	0 [0 1]	0.233
Pallor/leukoderma, median [1Q 3Q]	2 [2 3]	2 [2 3]	0.225
Purpuric lesions and excoriations, median [1Q 3Q]	0 [0 1]	1 [0 1.5]	0.191
Hyperkeratosis, median [1Q 3Q]	1 [0 2]	0 [0 1]	0.063

*Note*: In bold: Significant values.

Abbreviations: [1Q 3Q] interquartile range; VLS, vulvar lichen sclerosus.

^a^ 15 subjects in group A and 20 in group B were evaluable for dyspareunia.

### 3.2 Dropouts

During the study, three (10.3%) patients dropped out in group A and four (12.5%) patients in group B because they were either lost to follow‐up or discontinued the treatment for reasons other than local side effect occurrence. The rate of dropouts was not different between the two study groups (*P* = .99).

### 3.3 Effectiveness evaluations

Regarding the primary effectiveness endpoint, at the end of the treatment 31 patients (50.8% of the entire included population) achieved GSS = 0, 10 (16.4%) achieved GOS = 0, and 8 (13.1%) achieved a complete clearance of both symptoms and signs (GSS and GOS = 0) (Table 2). Considering group A and group B separately, the rates of patients who achieved complete resolution in symptoms (GSS), signs (GOS) or both (GSS and GOS) did not significantly differ between them.

**TABLE 2:** Rates of subjects who achieved clearance in global subjective score or global objective score or both at treatment completion

Variables	Study patients (total, n. 61)	Group A 24 week‐duration (total, n. 29)	Group B 12 week‐duration (total, n. 32)	*P*‐value
Global Subjective Score				.554
GSS = 0, n. (%)	31 (50.8)	16 (55.2)	15 (46.9)	
GSS >0, n. (%)	23 (37.7)	10 (34.5)	12 (40.6)	
Missing values	7 (11.5)	3 (10.3)	4 (12.5)	
Global Objective Score				.730
GOS = 0, n. (%)	10 (16.4)	4 (13.8)	6 (18.8)	
GOS >0, n. (%)	44 (72.1)	22 (75.9)	22 (68.7)	
Missing values	7 (11.5)	3 (10.3)	4 (12.5)	
GSS and GOS				.706
GSS and GOS = 0, n. (%)	8 (13.1)	3 (10.3)	5 (15.6)	
GSS and GOS >0, n. (%)	46 (73.8)	23 (79.4)	23 (71.9)	
Missing values	7 (13.1)	3 (10.3)	4 (12.5)	

*Note*: Missing values refer to the 7 subjects who dropped out (3 in group A and 4 in group B).

As regards the secondary effectiveness endpoints, the median scores of GSS, GOS and dyspareunia significantly decreased at the end of the treatment compared with baseline in the study subjects considered together (Table 3). A significant decrease in all these parameters was found in group A, whereas among group B patients median values decreased in a significant way for GSS and GOS. The variations in GSS, GOS and dyspareunia values were similar between group A and group B, according to Mann–Whitney test, as reported in Table 4.

**TABLE 3:** Changes in GSS, GOS and dyspareunia at study completion in the study subjects, considering the entire population and the two treatment groups

	Global Subjective Score (0–20)		Global Objective Score (0–12)		Dysparuenia (0–10)	
	Number	Median [1Q 3Q]	Number	Median [1Q 3Q]	Number	Median [1Q 3Q]
Study patients (total, n. 61)						
T0	61	12 [6 18]	61	4 [3 5]	35	6 [0 10]
T1	54	0 [0 4]	54	1 [1 2]	27	0 [0 5]
*P*‐value		**<.001**		**<.001**		**.021**
Group A, 24 week‐duration (total, n. 29)						
T0	29	14 [7 20]	29	4 [3 5]	15	10 [0 10]
T1	26	0 [0 6]	26	1 [1 2]	12	3 [0 6]
*P*‐value		**<.001**		**<.001**		**.049**
Group B, 12 week‐duration (total, n. 32)						
T0	32	12 [5.5 16]	32	4 [3 5.5]	20	2.5 [0 7.5]
T1	28	0 [0 6]	28	1 [1 2]	15	0 [0 3]
*P*‐value		**<.001**		**<.001**		.144

*Note*: Seven subjects dropped out (3 in group A and 4 in group B); 15 subjects in group A and 20 in group B were evaluable for dyspareunia at baseline; [1Q 3Q] interquartile; in bold: Significant values.

**TABLE 4:** Variation of GSS, GOS and dyspareunia, expressed in median values and inter‐quartile range, at the end of the treatment as compared with baseline

Variables		Study patients (total, n. 61)	Group A, 24 week‐duration (total, n. 29)	Group B, 12 week‐duration (total, n. 32)	*P*‐value
ΔGSS (0–20)	median [1Q 3Q]	−10 [−14‐5]	−10 [−17‐7]	−9.5 [−14‐5]	.504
ΔGOS (0‐12)	median [1Q 3Q]	−3 [−4‐2]	−3 [−4‐2]	−2 [−4‐1]	.103
Δdyspareunia (0‐10)	median [1Q 3Q]	0 [−5 0]	0 [−7 0]	0 [−5 0]	.843

Abbreviations: Δ, variation; GSS, Global Subjective Score; GOS, Global Objective Score; [1Q 3Q] interquartile range.

In Table 5 the rates of subjects who achieved GSS75, D75, and GOS75 at the end of the treatment are shown, considering the two groups separately. Comparing the two treatment groups, no significant differences were found for any of the assessed parameters.

**TABLE 5:** Rates of subjects who achieved an improvement from baseline of ≥75% in the GSS (GSS75), dyspareunia (D75) and GOS (GOS75) values

	Group A, 24 week‐duration (total, n. 29)	Group B, 12 week‐duration (total, n. 32)	*P*‐value
GSS75, n. (%)	18 (62.1)	22 (68.8)	.434
D75, n. (%)	4 (21.8)	7 (21.9)	.599
GOS75, n. (%)	13 (44.4)	10 (31.3)	.232

*Note*: Seven subjects dropped out (3 in group A and 4 in group B).

### 3.4 Tolerability assessment

At the 24‐ and 12‐week control visits, no patients reported significant side effects and none had to discontinue treatment because of them.

## 4 DISCUSSION

Distressing symptoms, chronic nature and progressive course are the main troublesome features of VLS. Two treatment phases may be scheduled in order to adequately face this disease, namely an initial acute or attack phase, and a subsequent long‐term, maintenance phase.^1^, ^16^ In the present study we aimed to assess the effectiveness and tolerability of an initial treatment phase, which was longer than the one currently recommended of 12 weeks duration. In particular, by comparing a 24‐week duration treatment with a 12‐week treatment, both scheduled with the same protocol, we were interested in assessing a possible advantage in achieving a complete disease clearance.

Considering the whole study population, regardless of treatment duration, about half of the included patients reached complete resolution of VLS‐related symptoms, namely itching and burning. About 16% of patients reached resolution of objective features and an even lower rate, that is 13%, achieved the clearance of the disease, which means complete healing of both symptoms and signs. These data were in line with previous reports.^15^ The most noteworthy finding was that the rates of subjects who achieved complete resolution of either symptoms or clinical features or both did not significantly differ between patients treated for 24 weeks and those treated for 12 weeks (Table 2). These results suggest that increasing the duration of corticosteroid therapy up to 24 weeks does not provide a therapeutic benefit in terms of complete cure of the disease, in comparison with a 12‐week standard treatment.

The secondary outcome assessments confirmed the great effectiveness of the potent corticosteroid in improving symptoms and, albeit to a lesser extent, clinical signs of VLS (Tables 3 and 5). Our results are in agreement with those available in the literature.^1^, ^4^, ^5^, ^6^, ^7^ The novel finding of our investigation was that 24‐week and 12‐week treatments did not differ, neither in terms of improvement in the median values of GSS and GOS at treatment completion (Tables 3 and 4) nor in the rates of patients achieving ≥75% improvement from baseline for all the study parameters (Table 5).

The occurrence of dropouts did not differ between group A and group B either. This may reflect a similar adherence to treatment, regardless of its duration. Thus, a higher rate of treatment discontinuation should not be expected by increasing the treatment course up to 24 weeks.

The safety profile was excellent for all the study patients and none of them complained of local side effects. This further supports the safety of topical corticosteroids in VLS treatment, even when they are applied for prolonged periods.^2^

The main limitation of this study is that it was not a randomized trial. Comparison between the different treatment durations was carried out matching two groups not generated by randomization. In spite of this, demographic and clinical features resulted well balanced between the two groups, except for dyspareunia (Table 1). The investigators who scored VLS signs were not blinded to treatment. Since univocal and validated methods to assess VLS severity as well as univocal definition of response are not available in the literature,^17^, ^18^ we chose to consider different outcome measures for assessing efficacy, in order to reduce the risk of methodological bias. In order to address the issue of patients' adherence to treatment, the rate of dropouts was considered whereas the true level of adherence was not objectively proved.

In spite of these limitations, this is the first study to compare 12‐ and 24‐week duration treatments in the attack therapy of VLS, with specific focus on clearance achievement. Based on our results, a 24‐week duration corticosteroid treatment does not seem to provide significant therapeutic advantages in comparison with standard 12‐week duration courses in terms of VLS clearance. It could be interesting to assess, through comparative studies, any therapeutic benefits from continuative treatments lasting even longer than 24 weeks. Since both tolerability and patient adherence did not differ between 24‐week and 12‐week duration treatments, physicians can prescribe either of the therapeutic protocols. In our opinion because of the chronic course of the disease, after an initial treatment phase of either 12‐ or 24‐week duration, a maintenance long‐term treatment phase is strongly recommended. In keeping with this, all patients who were judged as responsive at treatment completion, that is, who achieved an improvement of at least 75% in their signs and/or symptoms, underwent a proactive maintenance therapy. It consisted in the intermittent, low‐dose, namely twice a week, long‐term application, at least 52 weeks, to the previously affected areas of the same corticosteroid used in the active treatment phase, for maintaining remission and reducing risk of relapse.

## CONFLICT OF INTEREST

The authors declare no potential conflicts of interest.

## AUTHOR CONTRIBUTIONS

Monica Corazza: Design of the study, acquisition of data, interpretation of data, critical revision of the manuscript; Giulia Toni: Acquisition of data, critical revision of the manuscript; Giorgia Valpiani: Analysis and interpretation of data, drafting of the manuscript, critical revision of the manuscript; Chiara Morotti: Analysis and interpretation of data, drafting of the manuscript, critical revision of the manuscript; Alessandro Borghi: Design of the study, interpretation of data, drafting of the manuscript, critical revision of the manuscript. All authors read and approved the final manuscript.

## ETHICS STATEMENT

This study was approved by the Ethics Committee of University/Hospital of Ferrara, Italy (protocol n CE: 827/2020/Oss/AOUFe).

## Data Availability

The data that support the findings of this study are available on request from the corresponding author. The data are not publicly available due to privacy or ethical restrictions.

## References

[1] Kirtschig G, Becker K, Günthert A, et al. Evidence‐based (S3) guideline on (anogenital) lichen sclerosus. J Eur Acad Dermatol Venereol. 2015;29:e1‐e43. 10.1111/jdv.13136 26202852

[2] Lee A, Bradford J, Fischer G. Long‐term management of adult vulvar lichen sclerosus: a prospective cohort study of 507 women. JAMA Dermatol. 2015;151:1061‐1067. 26070005 10.1001/jamadermatol.2015.0643

[3] Chin S, Scurry J, Bradford J, Lee G, Fischer G. Association of topical corticosteroids with reduced vulvar squamous cell carcinoma recurrence in patients with vulvar lichen Sclerosus. JAMA Dermatol. 2020;156:813‐814. 32374363 10.1001/jamadermatol.2020.1074 PMC7203667

[4] Chi CC, Kirtschig G, Baldo M, Brackenbury F, Lewis F, Wojnarowska F. Topical interventions for genital lichen sclerosus. Cochrane Database Syst Rev. 2011;12:CD008240. 10.1002/14651858.CD008240.pub2 PMC7025763 22161424

[5] Lewis FM, Tatnall FM, Velangi SS, et al. British Association of Dermatologists guidelines for the management of lichen sclerosus, 2018. Br J Dermatol. 2018;178:839‐853. 29313888 10.1111/bjd.16241

[6] Virgili A, Borghi A, Toni G, Minghetti S, Corazza M. First randomized trial on clobetasol propionate and mometasone furoate in the treatment of vulvar lichen sclerosus: results of efficacy and tolerability. Br J Dermatol. 2014;171:388‐396. 24641522 10.1111/bjd.12910

[7] Borghi A, Corazza M, Minghetti S, Toni G, Virgili A. Continuous vs. tapering application of the potent topical corticosteroid mometasone furoate in the treatment of vulvar lichen sclerosus: results of a randomized trial. Br J Dermatol. 2015;173:1381‐1386. 26280156 10.1111/bjd.14074

[8] Funaro D, Lovett A, Leroux N, Powell J. A double‐blind, randomized prospective study evaluating topical clobetasol propionate 0.05% versus topical tacrolimus 0.1% in patients with vulvar lichen sclerosus. J Am Acad Dermatol. 2014;71:84‐91. 24704090 10.1016/j.jaad.2014.02.019

[9] Goldstein AT, Creasey A, Pfau R, Phillips D, Burrows LJ. A double‐blind, randomized controlled trial of clobetasol versus pimecrolimus in patients with vulvar lichen sclerosus. J Am Acad Dermatol. 2011;64:e99. 21353334 10.1016/j.jaad.2010.06.011

[10] Dalziel KL, Millard PR, Wojnarowska F. The treatment of vulval lichen sclerosus with a very potent topical steroid (clobetasol propionate 0.05%) cream. Br J Dermatol. 1991;124:461‐464. 2039723 10.1111/j.1365-2133.1991.tb00626.x

[11] Lorenz B, Kaufman RH, Kutzner SK. Lichen sclerosus. Therapy with clobetasol propionate. J Reprod Med. 1998;43:790‐794. 9777618

[12] Bracco GL, Carli P, Sonni L, et al. Clinical and histologic effects of topical treatments of vulval lichen sclerosus. A critical evaluation. J Reprod Med. 1993;38:37‐40. 8441129

[13] Cattaneo A, De Magnis A, Botti E, Sonni L, Carli P, Taddei GL. Topical mometasone furoate for vulvar lichen sclerosus. J Reprod Med. 2003;48:444‐448. 12856516

[14] Virgili A, Borghi A, Minghetti S, Corazza M. Mometasone fuoroate 0.1% ointment in the treatment of vulvar lichen sclerosus: a study of efficacy and safety on a large cohort of patients. J Eur Acad Dermatol Venereol. 2014;28:943‐948. 23879234 10.1111/jdv.12219

[15] Borghi A, Virgili A, Minghetti S, Toni G, Corazza M. Clearance in vulvar lichen sclerosus: a realistic treatment endpoint or a chimera? J Eur Acad Dermatol Venereol. 2018;32:96‐101. 28796915 10.1111/jdv.14516

[16] Borghi A, Corazza M. Novel therapeutic approaches and targets for treatment of vulvar lichen sclerosus. Curr Pharm Biotechnol. 2021;22:99‐114. 32416670 10.2174/1389201021666200516154310

[17] Sheinis M, Green N, Vieira‐Baptista P, et al. Adult vulvar lichen Sclerosus: can experts agree on the assessment of disease severity? J Low Genit Tract Dis. 2020;24:295‐298. 32205764 10.1097/LGT.0000000000000534

[18] Goodrum CA, Leighton PA, Simpson RC. Outcome domains in lichen sclerosus. Br J Dermatol. 2020;183:966‐968. 32471015 10.1111/bjd.19253

